# DNA Methylation Analysis of Dormancy Release in Almond (*Prunus dulcis*) Flower Buds Using Epi-Genotyping by Sequencing

**DOI:** 10.3390/ijms19113542

**Published:** 2018-11-10

**Authors:** Ángela S. Prudencio, Olaf Werner, Pedro J. Martínez-García, Federico Dicenta, Rosa M. Ros, Pedro Martínez-Gómez

**Affiliations:** 1 Department of Plant Breeding, CEBAS-CSIC, P.O. Box 164, Espinardo, 30100 Murcia, Spain; asanchez@cebas.csic.es (A.S.P.); pjmartinez@cebas.csic.es (P.J.M.-G.); fdicenta@cebas.csic.es (F.D.); 2 Department of Plant Biology, Faculty of Biology, University of Murcia, Espinardo, 30100 Murcia, Spain; werner@um.es (O.W.); rmros@um.es (R.M.R.)

**Keywords:** *Prunus*, flowering, bisulfite sequencing, genomics, epigenetics, breeding

## Abstract

DNA methylation and histone post-translational modifications have been described as epigenetic regulation mechanisms involved in developmental transitions in plants, including seasonal changes in fruit trees. In species like almond (*Prunus dulcis* (Mill.) D.A: Webb), prolonged exposure to cold temperatures is required for dormancy release and flowering. Aiming to identify genomic regions with differential methylation states in response to chill accumulation, we carried out Illumina reduced-representation genome sequencing on bisulfite-treated DNA from floral buds. To do this, we analyzed almond genotypes with different chilling requirements and flowering times both before and after dormancy release for two consecutive years. The study was performed using epi-Genotyping by Sequencing (epi-GBS). A total of 7317 fragments were sequenced and the samples compared. Out of these fragments, 677 were identified as differentially methylated between the almond genotypes. Mapping these fragments using the *Prunus persica* (L.) Batsch v.2 genome as reference provided information about coding regions linked to early and late flowering methylation markers. Additionally, the methylation state of ten gene-coding sequences was found to be linked to the dormancy release process.

## 1. Introduction

The almond tree (*Prunus dulcis* (Mill.) D.A. Webb), like the rest of the *Prunus* species, is a deciduous fruit tree that undergoes a cyclical process of flowering, sprouting, development, and winter rest, called dormancy. The dormancy state protects the plant from potential damage from cold weather during the winter [1,2]. The dormancy period is overcome when the tree accumulates sufficient chilling hours (the chilling requirement). After dormancy release, the tree is able to sprout and flower under favorable climatic conditions [3,4]. The study of molecular mechanisms leading to dormancy release and flowering is of great interest for almond breeding programs aiming to adapt new cultivars to specific growing areas [5,6]. The dormancy release process involves sensing environmental cues (such as temperature), signal transduction, and gene expression regulation to establish a suitable response according to the stimuli received [7,8]. Transcription reprogramming leading to dormancy release may thus be mediated by epigenetic mechanisms [9,10]. 

Epigenetics are chemical modifications affecting DNA or structural proteins (histones) within the chromatin. Two types of epigenetic modifications have been described: DNA methylation (5′ Methylated Cytosine, 5mC) in plants and histone Post-Translational Modifications (PTMs), which include the acetylation and methylation of histones [11,12]. 

Epigenetic changes are part of the transcriptional regulation machinery of genomes. The dynamic but heritable character of such modifications makes them interesting regulators mediating adaptive responses to environmental changes, such as seasonal cycles, and in the long term, climate change [13]. DNA methylation is associated with cell status stability and regulation of expression. DNA methylation occurs in three sequence contexts: CG and CHG, which are found in promoter and coding regions, and CHH (where H = A, C or T), found in non-coding regions and transposable elements (TEs) [14]. 

Several works have described the role of epigenetics in the regulation of dormancy in deciduous plant species. Santamaría et al. [15], for instance, described a methylation decrease concomitant with H4 deacetylation and the progress of dormancy release in *Castanea sativa* Mill. In peach (*Prunus persica*), de la Fuente et al. [16] identified a genome-wide pattern of the PTM Trymethylation of Histone 3 on Lis (K) residue 27 (H3K27me3) during bud dormancy release, and Lloret et al. [17] found a relationship between gene expression, PTMs, and sorbitol synthesis during bud dormancy progression and release. Rothkegel et al. [18] showed that DNA methylation is one of the mechanisms participating in the regulation of MADS-box (MCM1-AGAMOUS-DEFICIENS-SRF) genes controlling bud dormancy in sweet cherry (*Prunus avium* L.). In apple (*Malus domestica* (Suckow) Borkh.), genome methylation patterns have been linked to chilling acquisition during dormancy [19]. In the case of almond, preliminary results from the transcriptome sequencing of non-dormant and dormant flower buds showed differential expression in a DNA methyltransferase gene and in the *S-ADENOSYL METHIONINE SYNTHETASE* gene responsible for the synthesis of the molecule SAM (S-adenosyl methionine), which donates the methyl group to the DNA molecule [20]. In addition, DNA methylation phenomena have also been associated with floral self-incompatibility [21] and with bud falling phenomena [22] in this species. 

Genome-wide analysis of DNA methylation can be done by bisulfite sequencing, which uses Next Generation Sequencing (NGS) to analyze digested and bisulfite-treated DNA samples. The epi-Genotyping by Sequencing (epiGBS) technique was developed to represent a small part of the genome for cost-effective exploration and comparative analysis of DNA methylation and genetic variation in hundreds of de novo samples. Furthermore, this method makes it possible to genotype samples without a prior reference genome [23]. 

The objective of this work was to analyze the DNA methylation status of almond flower buds using epi-GBS for the first time. For this purpose, we evaluated dormant and non-dormant flower buds from two almond genotypes with different chilling requirements and flowering times using the epi-GBS protocol. 

## 2. Results

### 2.1. Evaluation of the Quality of the Epi-GBS Analysis

**Figure 1:**
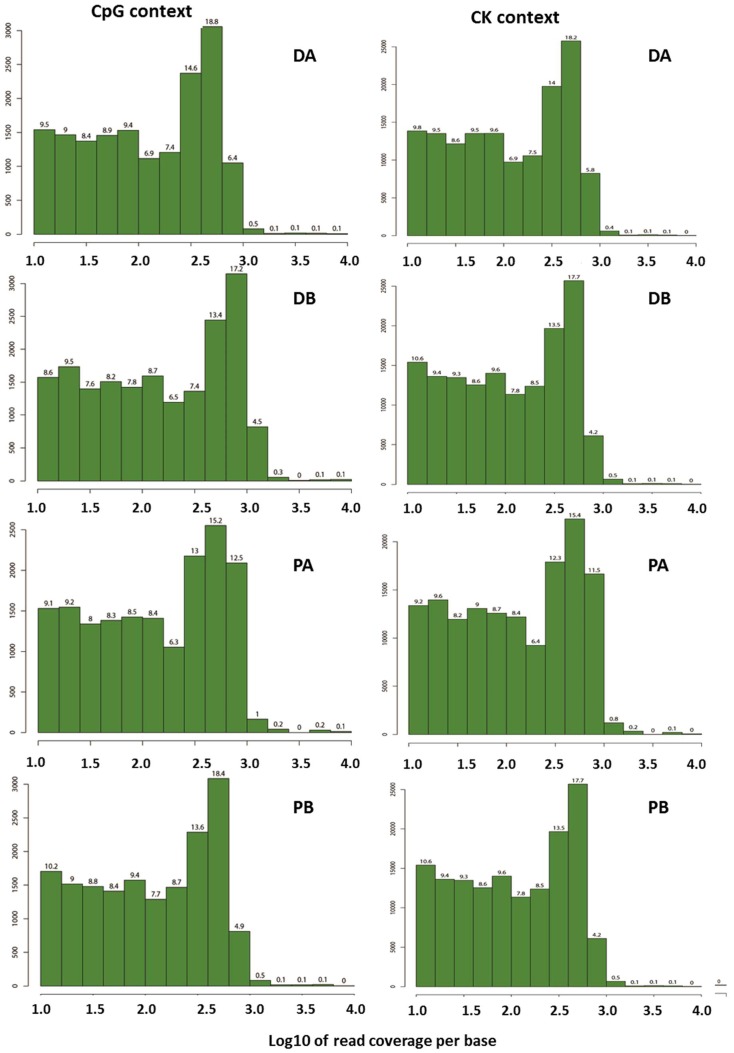
Read coverage of the samples tested in the CpG and CK (including CHG and CHH) contexts during the 2015–2016 season. “D” = ‘Desmayo Largueta’, “P” = ‘Penta’, “A” = dormant bud stage, “B” = non-dormant bud stage.

We sequenced 9518 fragments (about a 1244 kb size) and identified 7317 methylated or unmethylated fragments. Furthermore, we were able to reconstruct the original sequence of 4377 fragments. The total length of the “mock genome” obtained by merging the reconstructed fragments was 662,458 bp. Regarding the quality of this epi-GBS analysis, the absence of a secondary peak towards the right of the read coverage histograms shows that the data do not suffer from PCR duplication bias in either year (Figure 1; data from the flower buds sampled in 2015–2016). These read coverage results show the uniformity of the reads and correct PCR amplification (good quality) around the whole genome in both contexts and seasons of study.

**Figure 2:**
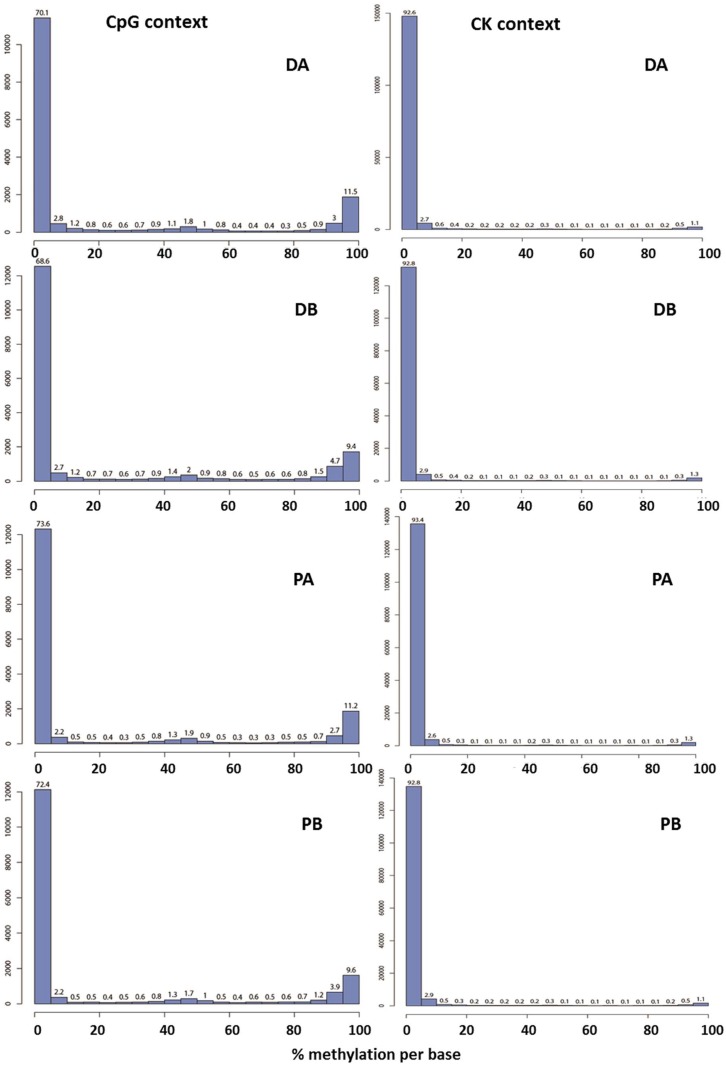
Percentage of DNA methylation of the samples tested in the CpG and CK (including CHG and CHH) contexts during the 2015–2016 season. “D” = ‘Desmayo Largueta’, “P” = ‘Penta’, “A” = dormant bud stage, “B” = non-dormant bud stage.

The histograms of CpG methylation showed that roughly 70% to 75% of the cytosine positions in the CpG context of the mock genome were unmethylated, around 10% of the positions were completely methylated and the remaining positions were partially methylated to varying degrees in both seasons of study (Figure 2; data from 2015–2016 season). 

**Figure 3:**
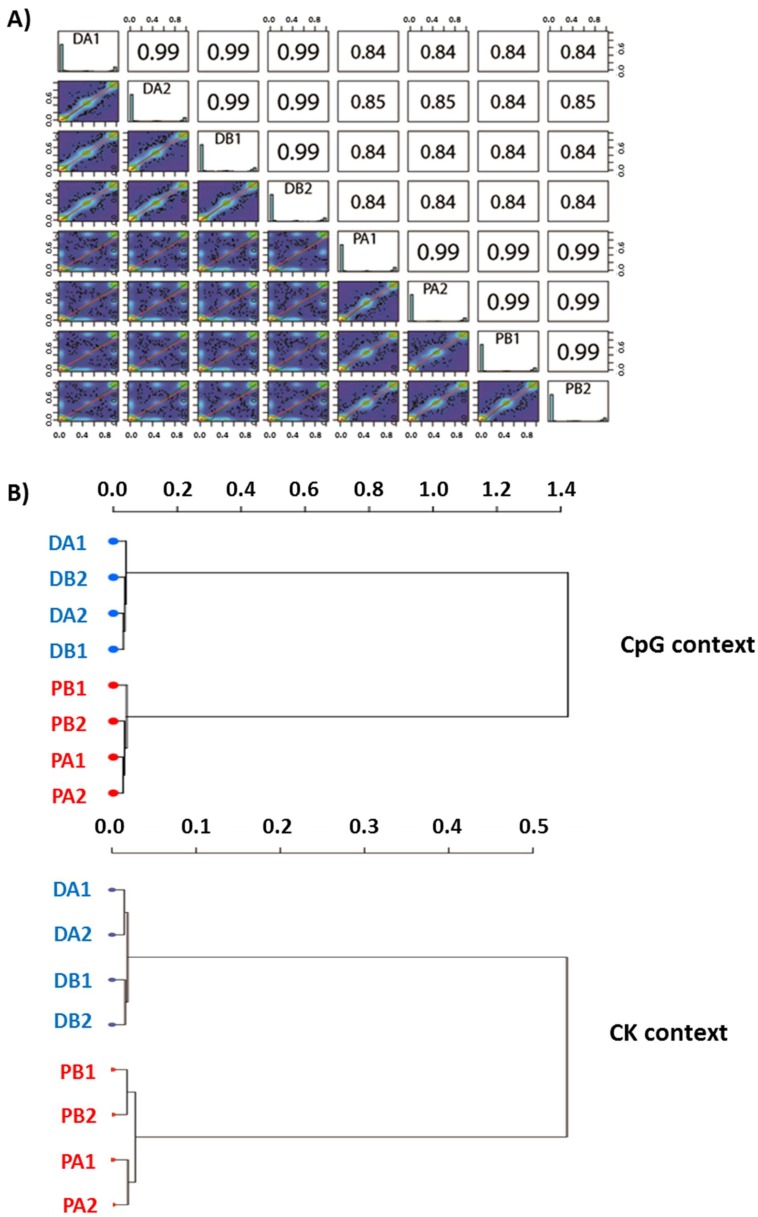
(**A**) Almond correlations and (**B**) clustering analysis of the methylated fragments in both CpG and CK contexts. “D” = ‘Desmayo Largueta’, “P” = ‘Penta’, “A” = dormant bud stage, “B”=non-dormant bud stage. “1” samples are from the 2015–2016 season and “2” samples are from the 2016–2017 season.

Furthermore, the correlation analyses clearly show that samples of the same variety cluster together independently of the developmental stage. The Pearson’s correlation coefficient was constantly 0.99 in comparisons within each variety and in the range of 0.84 to 0.85 in comparisons between samples of different varieties (Figure 3A). These results were also corroborated by clustering analysis, in which samples belonging to the same variety were close together while the two varieties were separated by long branches (Figure 3B). The DNA methylation pattern is generally variety-dependent rather than dormancy-dependent.

### 2.2. Differentyally Methylated Genes Detected

Quantitative analysis showed that 7317 different fragments were methylated in at least one sample: 5109 ‘Cs’ were methylated in ‘Desmayo Largueta’ A samples; 5089 ‘Cs’ were methylated in ‘Desmayo Largueta’ B samples; 4955 ‘Cs’ were methylated in ‘Penta’ A samples; and 5003 ‘Cs’ were methylated in ‘Penta’ B samples.

Table 1 shows that the number of differentially methylated fragments (DMFs) detected was variable depending on the comparison performed. Furthermore, a total of 677 DMFs were found between ‘Desmayo Largueta’ and ‘Penta’ genotype samples in all stages analyzed. However, when comparing dormancy state samples (A and B), 23 DMFs were found between ‘Desmayo Largueta’ stage A and ‘Desmayo Largueta’ stage B samples and 48 DMFs were found between ‘Penta’ stage A samples and ‘Penta’ stage B samples. Of those DMFs, ten were common between ‘Desmayo Largueta’ and ‘Penta’ in the A to B stage comparison. The DMFs were divided into hypermethylated or hypomethylated categories using ‘Penta’ or ‘stage B’ samples as the reference. The fragment sequences are included in Table S1.

More than 99% of the identified DMFs were mapped on the *Prunus persica* v2.1 genome (Table S2), and those located between 2 kb upstream and 1 kb downstream from the gene coding sequences were selected for subsequent annotation analysis. The number of differentially methylated genes (DMGs) thus identified is shown in Table 2 and Table 3. 

DMGs were classified according to their position with respect to the fragment-mapping region. The most frequently mapped fragments were those within gene regions (“inside” DMGs) followed by the 5′regulatory regions (“downstream” DMGs) and, finally, in the 3′ regions (“upstream” DMGs). 

Data shown in Table 2 indicate that DMGs were found as hypermethylated in ‘Desmayo Largueta’ samples (in both the A and B stages) to a greater extent than in ‘Penta’ samples (423).

**Figure 4:**
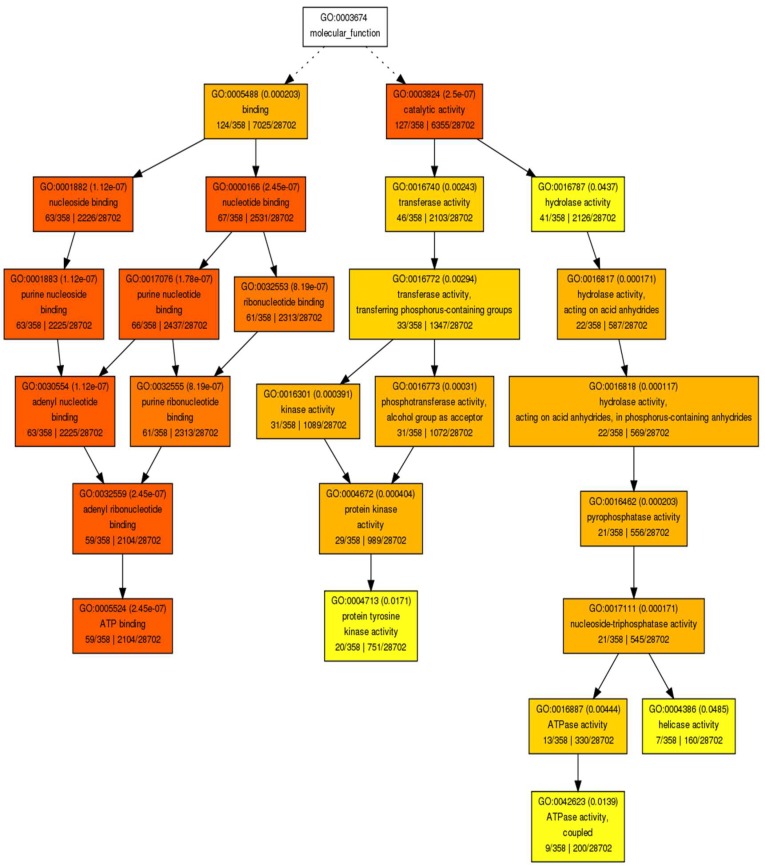
GO terms of the “Molecular function” category represented in genes identified as hypermethylated in ‘Desmayo Largueta’ flower buds in both the “A” = dormant bud stage and “B” = non dormant bud stage.

We found enriched hypermethylated genes in ‘Desmayo Largueta’ flower bud samples in the following processes related to primary metabolism in the “biological function” GO (Gene Ontology) category: amino-acid and carbohydrate synthesis and protein phosphorylation (Figure S1). ATP binding and protein kinase and phosphatase activity were the two main “molecular function” GO terms found (Figure 4).

In a gene-level analysis, the following candidate genes appeared as hypermethylated in ‘Desmayo Largueta’ flower bud samples: genes related to transcription regulation, including transcription factors (Prupe.1G395600, Prupe.5G088700, Prupe.6G343100); genes linked to RNA-mediated silencing (Prupe.7G221200); genes linked to chromatin remodelling (Prupe.8G221300, and LATE ELONGATED HYPOCOTYL (LHY), encoded by Prupe.2G200400); and, especially, genes involved in the auxin response (Prupe.1G000200, Prupe.1G067400, Prupe.7G048400) and AUXIN RESPONSE FACTOR (ARF) signal transduction (Prupe.3G010900, Prupe.5G217700, Prupe.7G228800). We also identified DNA repair proteins, such as those encoded by Prupe.1G510000, Prupe.2G013900, Prupe.3G029600, Prupe.3G16000 and Prupe.5G066100. Finally, proteins participating in oxidoreduction processes, such as LATE EMBRYOGENESIS ABUNDANT (LEA) proteins encoded by Prupe.4G026900 and Prupe.4G02700, also appeared as hypermethylated in ‘Desmayo Largueta’ flower bud samples (Table S3). 

**Figure 5:**
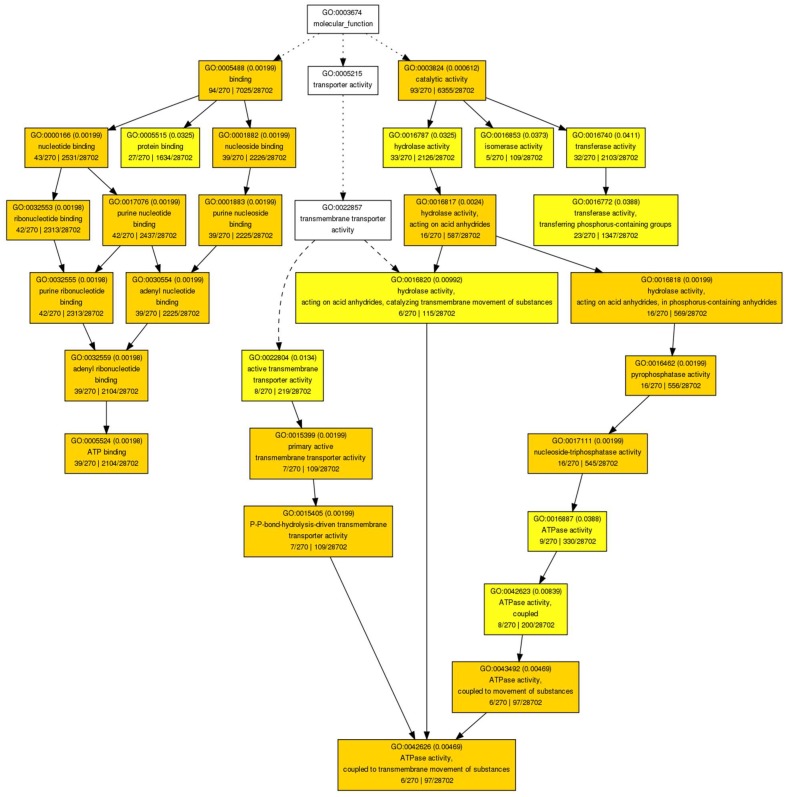
“Molecular function” GO terms represented in genes identified as hypomethylated in ‘Desmayo Largueta’ flower buds in both the “A” = dormant bud stage and “B” = non dormant bud stage.

Regarding the hypomethylated genes in the ‘Desmayo Largueta’ samples, we found cellular protein localization within the “biological function” GO category (Figure S2). ATP-coupled transmembrane transport and ATP-binding activity, on the other hand, appeared in the “molecular function” GO category (Figure 5). 

We were able to identify a wide range of DNA-binding proteins encoded by the hypomethylated genes in the ‘Desmayo Largueta’ samples: histone methyltransferases (encoded by Prupe.1G050800 and Prupe.7G271600); the transcription factor NUCLEAR FACTOR-Y (NF-Y) (encoded by Prupe.2G47600); DNA topoisomerases (encoded by Prupe.1G173400 and Prupe.1G173500); and FAR-RED IMPAIRED RESPONSE 1 (FAR1) (Prupe.1G196400). Interestingly, a single gene coding for a HYDROPHOBIC SEED PROTEIN (HSP) also appeared as hypomethylated in the ‘Desmayo Largueta’ samples. 

### 2.3. Differentyally Methylated Genes Related to Bud Dormancy

More identified DMGs were found as hypermethylated in stage A samples (dormant buds) than in stage B samples (non-dormant buds) (Table 3). Furthermore, just one hypomethylated gene could be functionally annotated, and it was mapped in the 3’ regulatory region of the gene Prupe.4G277200, which encodes for a REGULATION OF CHROMOSOME CONDENSATION (RCC1) protein (Table 4).

Common stage A hypermethylated genes coded for a MITOGEN-ACTIVATED PROTEIN (MAP)-kinase and a phosphatase (Prupe.4G270800 and Prupe.1G287200); an LEUCINE RICH REPEAT-TOLL INTERLEUKIN 1 RECEPTOR (LRR-TIR) apoptotic ATPase associated with disease resistance (Prupe.3G130700); a GDSL (Gly, Asp, Ser and Leu motif) lipase (Prupe.6G307900); an Nt-C2 family protein (Prupe.2G074400); and a Glycerophosphatidylinositol (GPI) anchor synthase (Prupe.2G019300). The Prupe.1G125600 gene was annotated, but its protein function is unknown (Table 4). Moreover, genes coding for VACUOLAR PROTEIN SORTING 1 (VPS1) proteins were detected as hypermethylated in stage A in both ‘Desmayo Largueta’ and ‘Penta’. VPS1 genes corresponded to Prupe.3G026400 in ‘Desmayo Largueta’ samples and Prupe.2G029500 in ‘Penta’ samples.

Three additional LRR-TIR apoptotic ATPases were identified as encoded by hypermethylated genes in ‘Desmayo Largueta’ stage A samples (Table 4). Other genes that were found coded for defense proteins, a phosphatase, a cornichon protein associated with cell polarity and a Cytochrome P-450 (CytP450) protein member. On the other hand, hypermethylated genes in ‘Penta’ stage A samples coded for the ASSEMBLY PROTEIN 180 (AP180) clathrin assembly protein, the Tryptophan-Aspartic acid-Sterile Alpha Motif domain containing protein (WDSAM1) ubiquitination protein, lipases, a glycosyl hydrolase and a FCF2 rRNA processing protein recently described in yeast (Table 4). 

## 3. Discussion

Using our epiGBS variant as a first approach is a less expensive technique than complete GBS with highly accurate results. Furthermore, without the sequenced genome of the species, it is easier to perform bioinformatic analysis with well-defined fragments obtained by epiGBS. In this work, a conversion with bisulfite and a subsequent sequencing were performed to evaluate the 5mC variants of the samples analyzed. Subsequently, using bioinformatic analysis, these differentially methylated regions were mapped in the reference genome.

Applying epi-GBS to ‘Desmayo Largueta’ and ‘Penta’ flower bud gDNA samples provided data about methylation (5mC) variants depending on the genotype and dormancy state of the flower buds. Quality evaluation of the analysis showed that more than 90% of all cytosine positions were completely unmethylated and that only 1.0% to 1.3% of the positions were completely methylated, with higher methylation in the CpG context. These results agree with previous results in different plant species indicating that CG methylation is the typical genomic region for DNA methylation with less methylation abundance in the CHG and CHH contexts [23]. As a result of the specificity of the methylome of plants with respect to that of animals, we adapted bisulfite conversion methods to allow for correct analysis in plants for all cytosine contexts [24]. 

DNA methylomes have now been analyzed in many plants species, including Arabidopsis, rice, maize, and tomato. DNA methylation results in these species indicate that the distribution of methylation marks across the genomes is generally conserved, although variations can be observed between species depending on several factors, including transposon abundance and genome size [25]. In agreement with our results, polymorphism (5mC variants) can be observed by comparing different genotypes or even accessions within the same species. In addition, recent results evaluating methylomes from 1,227 different accessions of *Arabidopsis* distributed worldwide have shown important polymorphisms between accessions [26].

It is interesting to note the high degree of differential methylation that seems to be fixed between the two almond genotypes analyzed. This fact is of practical importance in cultivar improvement for developing epigenetic markers based on methylation variants and taking into account the high flexibility of methylation patterns in relation to external signals in order to identify markers based on methylation polymorphisms. In contrast to standard sequencing, bisulfite sequencing makes it possible to obtain information that conditions the phenotype. As a consequence, knowing the methylation state might help us understand the genetic determinism of important agronomic traits more deeply. Although the methylation patterns are highly variable in response to different external factors, the markers that we have detected in our almond genotypes are conserved in different stages of development and in different years and can therefore be considered as stable and conserved epigenetic marks.

In this study, data showed important differences between genotypes, which displayed different phenotypes in terms of breeding traits (chilling requirements for dormancy release, flowering and ripening times, almond production and almond characteristics) (Table 1 and Table 2). It is remarkable that more hypermethylated than hypomethylated fragments were identified in stage A (dormant flower buds) almond samples in both genotypes (Table 3). This is concordant with the general decrease in 5mC during dormancy progression in *C. sativa* [15]. On the other hand, the most frequently mapped fragments were within the gene regions (“inside” DMGs), followed by the 5′regulatory regions (“downstream” DMGs) and, in last place, in the 3′ regions (“upstream” DMGs). According to Vining et al. [27], 5mC in promoters and gene body parts is related to a repressed state of chromatin, a condition that inhibits the accessibility of the transcriptional machinery.

Among the genes found as hypermethylated in ‘Desmayo Largueta’ with respect to ‘Penta’ flower buds (Table S3), ARF transcription factors were highly represented. Its known that the expression of genes like ARFs involved in the auxin response are subjected to epigenetic regulation [9,28], and ARF transcriptional regulation is required for developmental processes like germination [29]. Accordingly, Zhang et al. [30] observed a flowering delay in Arabidopsis when *ARF6* and *ARF8* were repressed. Nonetheless, the reason underlying the hypermethylated state of genes participating in the auxin response pathway in both dormant and non-dormant flower buds of the early flowering genotype ‘Desmayo Largueta’ has yet to be unraveled. 

Another hymermethylated gene in ‘Desmayo Largueta’ flower bud samples was a member of the LEA family (Table S3). LEA proteins are involved in osmoprotection, which is activated in response to low temperatures [31]. When hypermethylated, this gene showed a repressed state of expression, although in low chilling requirement cultivars like ‘Desmayo Largueta’, osmoprotection would not be so necessary or may be regulated in a different way. The LEA gene family has been characterized by Du et al. [32] in *Prunus mume* (Siebold) Siebold & Zucc., and differential expression has been identified during bud dormancy in this species [33].

The LHY protein, on the other hand, is a well-described flowering time regulator in response to the photoperiod [34,35], and the gene network controlling this trait has been studied [36]. ‘Desmayo Largueta’ is a low-chill cultivar whose dormancy period takes place under short photoperiod conditions such as the experimental conditions of this work. It would be interesting to study LHY behavior during dormancy progression in different almond cultivars.

The methylation variants observed may be associated with evolutionary changes related to each genotype’s features [37]. It will be interesting to distinguish which variants are related to traits of agronomic interest in order to explore adaptive mechanisms to the environment [38]. Recently, for instance, Garg et al. [39] identified conserved methylation polymorphisms distributed throughout rice varieties with different responses to drought resistance.

We found other hypermethylated genes in dormant (A) flower buds with respect to dormancy released (B) flower buds, including a MAP kinase (MAPK) and a phosphatase (Prupe.1G287200). MAP kinases and phosphatases have been found to participate in the initial response to cold induced by an increase in Ca2+ [31]. Furthermore, MAPK3 has been shown to be a central regulator of seed dormancy in barley [40]. Regarding the other genes hypermethylated in the A state, LRR-TIR apoptotic ATPases may be activated in a type of programmed cell death (PCD) called developmental cell death (DCD), leading to a differentiation of cells after dormancy release, as occurs in floral morphogenesis or in the pollen tube [41,42]. Nt-C2 and VSP1 proteins, on the other hand, are involved in vesicular trafficking from the cell membrane, and this process has been linked to cell wall differentiation and appears to be important in the dormancy release process [43,44]. Finally, GPI anchoring (a post-translational modification of proteins consisting of glycosylation) proteins are involved in intercellular signaling, as occurs in flowering transition as shown in *Populus* genus by Rinne et al. [45].

## 4. Materials and Methods 

### 4.1. Plant Material and Experimental Design

**Figure 6:**
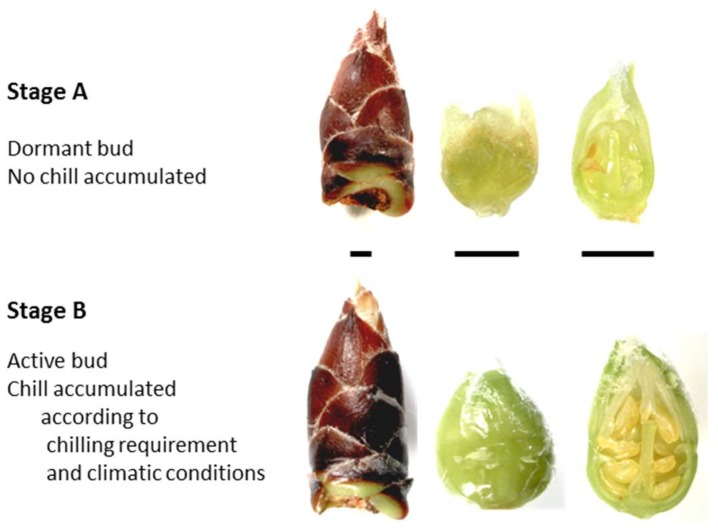
Plant material from ‘Desmayo Largueta’ and ‘Penta’ almond cultivars. Flower buds in the dormant state (**A**) and after dormancy release (**B**) according to Felipe [41]. Scale bars represent 1 mm in each case.

We used flower buds from ‘Desmayo Largueta’, a traditional almond cultivar with very low chilling requirements and an extra-early flowering time, and ‘Penta’, a cultivar released from the CEBAS-CSIC Almond Breeding Program (Murcia, South-East Spain) with high chilling requirements and an extra-late flowering time. The plant material consisted of flower buds at stages A (dormancy phase) and B (after dormancy release) that were referenced to the phenological stages described by Felipe [46] (Figure 6). Dormancy release evaluation was performed by the forcing method according to Prudencio et al. [6]. Almond flower buds were picked from the experimental field of CEBAS-CSIC during two seasons of study: 2015–2016 and 2016–2017.

### 4.2. Epi-GBS Protocol

Every sample (‘Desmayo Largueta’ stage A, ‘Penta’ stage A, ‘Desmayo Largueta’ stage B, and ‘Penta’ stage B from the first and second season of study) consisted of a pool of ten flower buds. Genomic DNA was extracted from each sample following the method described by Doyle and Doyle [47]. The DNA samples were quantified using Qubit (Thermo Fisher Scientific, Alcobendas, Spain) and diluted to 1 µg in 100 µL. A total of 20 µL was digested using *PstI* restriction enzyme. Adaptors consisting of barcoded oligos were ligated to every sample (Table S4). Non-phosphorylated hemimethylated adapters were used to reduce costs. Fragmented samples (libraries generated by restriction) were pooled and purified and subsequently subjected to nick translation with C-dNTPs (Zymo Research, Irvine, CA, USA) and 7.5 µL of DNA PolI (NEB, Ipswich, MA, USA) in NEB buffer 2. An EZ DNA Methylation-Lightning kit (Zymo Research) was used for bisulfite treatment, and fragments were selected by size with a Thermo Scientific Size Selection kit (Thermo Fisher Scientific). Libraries were amplified using the Kapa HiFi HotStart Uracil+ ReadyMix (Roche, Barcelona, Spain) and purified with Magjet NGS Cleanup (Thermo Fisher Scientific). Paired-end Illumina 2500 reads (2 × 100 bases) were generated by Macrogen (Seoul, Korea) [23].

### 4.3. Bioinformatic Analysis of DNA Methylation 

The process_radtags program of the Stacks 1.48 pipeline [48]. Checking the integrity of the restriction site was disabled with the “-disable_rad_check” option and quality filtering with the default settings was disabled with the exception the rad_check. This was necessary because the bisulfite treatment changes unmethylated cytosines in the recognition sequence of *PstI*, and, as a result, checking the restriction cut site would filter out all fragments. The ustacks program of the pipeline was used to align the fragments into perfectly matching stacks. The default settings were used with the exception of -M, which was set to 4 in order to increase the maximum distance (in nucleotides) between stacks. Finally, cstacks was used to build a catalog of consensus loci. A custom C program was used for the reconstruction of the original sequences of the fragments by comparing the reads with origins in the “Watson” and “Crick” strands of the genomic DNA. The reconstructed DNA fragments were merged by another custom C program to produce one continuous “mock genome”. Bismark_v0.19.0 [49] was used to align the original fragments to the mock genome and to extract the methylation information. The Bismark coverage reports were used as input for the methylKit R package [50]. A methyl kit was used to elaborate histograms of C-methylation and coverage and to assess sample similarity and correlation using the default settings. For the hierarchical clustering of the samples, dist was set to “correlation” and method to “ward”. Finally, we used the calculate DiffMeth function of a MethylKit to search for differentially methylated cytosines with the settings difference = 25, qvalue = 0.01. We looked for both hypermethylated and hypomethylated bases setting type = hyper and = hypo, respectively. The positions of the differentially methylated cytosines were extracted from the MethylKit files. Another custom made C-program was used to identify the original fragments where these differentially methylated cytosines were located. 

### 4.4. Gene Finding and Annotation 

**Figure 7:**
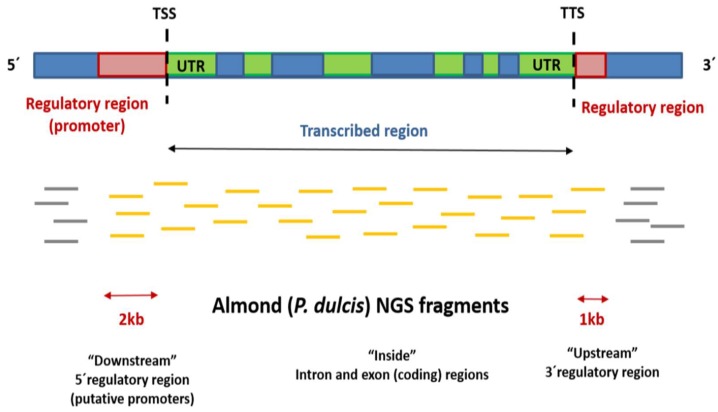
Schematic representation of a plant gene and classification of almond (*Prunus dulcis*) DMFs mapped in the *P. persica* genome. DMFs mapping from 2 kb upstream TSS to 1 kb downstream TTS were selected for functional annotation (fragments colored in orange). DMFs were classified according to the gene position—downstream, inside, or upstream—with respect to the fragment-mapping region. Fragments mapped in intergenic regions (colored in grey) were discarded as putative gene regulatory regions. **TSS:** Transcriptional Start Site, **UTR:** Untranslated Transcription Region, **TTS:** Transcriptional Terminal Site. Exons and introns within the transcribed region are colored in blue and green, respectively.

The sequence of each fragment was mapped against the *P. persica* reference genome (v2.0) [51] with Gmap [52]. Two different output files, in the gff3 and SAM format, were obtained. The gff3 ouput files were processed to extract the boundary coordinates (start and end positions) of each hit using command line tools. After that, the boundary coordinates were used by a second custom python script to retrieve three different categories of annotations based on gene locations on the *P. persica* reference genome: upstream and downstream genes (in a size window of 10,000 bp) and “inside genes” (fragments within gene sequence). Finally, SAM format files were processed using a custom python script to extract the alignment information (number of exons, percentage of coverage, percentage of identity, and amino acid changes). Functional annotation of genes selected by distance to the mapped fragment was carried out using AgriGO software using Singular Enrichment Analysis (SEA), and Fisher’s test [53] (Figure 7). 

## 5. Conclusions

In this study, we applied the epi-GBS protocol to almond (*P. dulcis*) DNA samples for the first time. The technical potential is evident in the discovery of epigenetic variants, based on 5mC, that are genotype-dependent. According to the results obtained, the DNA methylation (5mC) pattern is generally genotype-dependent rather than dormancy state-dependent. Comparative DNA methylation studies of both almond varieties released from breeding programs and traditional varieties will surely contribute to our knowledge of methylation variants and provide candidate epialleles linked to agronomic traits. Such polymorphisms can be screened in large populations using NGS to confirm the locus methylation state associated with a given character of interest. In spite of coverage limitation, we were able to identify genes whose DNA methylation state changed between the dormant and active state of the flower buds. This was possible in both the traditional early-flowering genotype ‘Desmayo Largueta’ and the extra-late-flowering genotype ‘Penta’ from the CEBAS-CSIC Almond Breeding Program. Furthermore, common genes arose from the analysis. In the future, it would be interesting to improve the technique coverage to obtain a greater representation of the genome. Ultimately, the results will be an essential complement to RNAseq experiments in bud dormancy progression.

## Figures and Tables

**Table 1:** Number of differentially methylated fragments (DMFs) detected by epi-Genotyping by Sequencing (epi-GBS) according to sample comparisons.

Differentially Methylated Fragments		
D–P genotype comparison		
Hypo (<)	Hyper (>)	Stages
307	370	A and B
677 DMFs		
A–B stage comparison		
Hypo (<)	Hyper (>)	Genotype
3	20	D
21	27	P
3	7	D and P
10 DMFs		

“D” = ‘Desmayo Largueta’, “P” = ‘Penta’; “A” = dormant bud stage, “B” = non-dormant bud stage.

**Table 2:** The number of differentially methylated genes (DMGs) identified from sequenced fragments mapping onto the *Prunus persica* genome (v2.1).

Differentially Methylated Genes			
Methylation State	Gene Position	Gene Hits	Genes Identified
Hypermethylated	Upstream	36	
	Inside	291	
	Downstream	134	
Total		461	423
Hypomethylated	Upstream	19	
	Inside	201	
	Downstream	80	
Total		300	281
Equally-methylated	Upstream	6	
	Inside	41	
	Downstream	8	
Total		55	27
Total DMGs		816	731

The methylation state refers to the number of 5′ Methylated Cytosines (5mCs) in ‘Desmayo Largueta’ samples compared to ‘Penta’ samples. The category “equally methylated” refers to genes whose number of 5mCs was the same between samples but in which the 5mCs were located in different fragment positions. The gene position is based on gene orientation with respect to the fragment-mapping region (“upstream”, “inside”, and “downstream”).

**Table 3:** The number of differentially methylated genes between the A and B dormancy states of flower buds, identified from sequenced fragments mapped to the *P. persica* genome (v2.1).

Methylation State	Gene Position	Differentially Methylated Genes		
		‘Desmayo Largueta’	‘Penta‘	Common
Hyper-methylated	Upstream	7	3	1
	Inside	5	2	2
	Downstream	5	9	4
Total		17	14	7
Hypo-methylated	Upstream	1	-	-
Total		18	14	7

The methylation state refers to the number of 5mCs in stage A (dormant buds) samples with respect to the number found in stage B (non-dormant buds) samples. The gene position is based on gene orientation with respect to the fragment-mapping region.

**Table 4:** Hypermethylated genes identified in ‘Desmayo Largueta’ (D) and ‘Penta’ (P) stage A flower buds compared to those found in stage B flower buds (Chromosome, Fragment ID, Prupe.ID, Functional annotation).

FragmentID	Comparison	Chromosome	Gene Position	Prupe.Gene Code
fragment1289	AB	Pp01	Downstream	Prupe.1G287200
fragment1294	AB	Pp01	Upstream	Prupe.1G125600
fragment3735	PAPB	Pp01	Downstream	Prupe.1G099900
fragment32	AB	Pp03	Downstream	Prupe.3G130700
fragment341	AB	Pp06	Downstream	Prupe.6G307900
fragment797	AB	Pp02	Inside	Prupe.2G074400
fragment1708	DADB	Pp02	Inside	Prupe.2G019300
fragment4206	PAPB	Pp02	Inside	Prupe.2G019300
fragment341	DADB	Pp02	Downstream	Prupe.2G031100
fragment255	DADB	Pp02	Upstream	Prupe.2G053300
fragment92	DADB	Pp02	Inside	Prupe.2G057100
fragment92	DADB	Pp02	Inside	Prupe.2G057800
fragment797	DADB	Pp02	Upstream	Prupe.2G074300
fragment3707	PAPB	Pp04	Downstream	Prupe.4G186400
fragment4154	PAPB	Pp04	Downstream	Prupe.4G253800
fragment238	AB	Pp04	Downstream	Prupe.4G270800
fragment3299	PAPB	Pp06	Downstream	Prupe.6G307900
fragment1289	DADB	Pp02	Inside	Prupe.2G146000
fragment341	DADB	Pp03	Upstream	Prupe.3G026400
fragment797	DADB	Pp03	Downstream	Prupe.3G130700
fragment2263	DADB	Pp05	Inside	Prupe.5G036900
fragment157	PAPB	Pp06	Upstream	Prupe.6G014500
fragment1289	DADB	Pp05	Downstream	Prupe.5G038500
fragment483	PAPB	Pp02	Upstream	Prupe.2G039500
fragment507	DADB	Pp06	Inside	Prupe.6G097800
fragment1708	DADB	Pp06	Upstream	Prupe.6G331300
fragment2727	DADB	Pp06	Upstream	Prupe.6G331300
fragment849	PAPB	Pp01	Downstream	Prupe.1G105700

## Supplementary Materials

The supplementary materials can be found at http://www.mdpi.com/1422-0067/19/11/3542/s1. Figure S1. GO terms of the “Biological Function” category represented in genes identified as hypermethylated in ‘Desmayo Largueta’ flower buds, in both the A and B dormancy states, compared to ‘Penta’ flower buds. Figure S2. GO terms of the “Biological Function” category represented in genes identified as hypomethylated in ‘Desmayo Largueta’ flower buds, in the both A and B dormancy states, compared to ‘Penta’ flower buds. Table S1. Total NGS fragments identified by Fragment ID. Table S2. Percentage of DMFs mapped in the *Prunus persica* genome (v2.1), Mapping region, Coverage, Percent identity, Number of exons, Amino acid changes, Comparison and Category. Table S3. Hypermethylated, Hypomethylated and Equally-methylated genes detected in ‘Desmayo Largueta’ flower buds with respect to ‘Penta’ flower buds in both the A and B stages (Chromosome, Fragment ID, Prupe.ID, Functional annotation). Gene list for GO annotation. Table S4. Sample barcodes. “D” = ‘Desmayo Largueta’, “P” = ‘Penta’, “A” = dormant bud stage; “B” = non-dormant bud stage. DNA samples with number “1” correspond to the 2015–2016 season, and those with number “2” correspond to the 2016–2017 season.

## Abbreviations

5mC	5′ Methylated Cytosine
AP180	ASSEMBLY PROTEIN 180
ARF	AUXIN RESPONSE FACTOR
CytP450	Cytochrome P-450
DMF	Differentially Methylated Fragment
DMG	Differentially Methylated Gene
epiGBS	Epi-Genotyping By Sequencing
FAR1	FAR-RED IMPAIRED RESPONSE 1
GDSL	Gly, Asp, Ser, and Leu motif
GO	Gene Ontology
GPI	Glycerophosphatidylinositol
H3K27me3	Trymethylation of Histone 3 on Lis (K) residue 27
HSP	HYDROPHOBIC SEED PROTEIN
LEA	LATE EMBRYOGENESIS ABUNDANT
LHY	LATE ELONGATED HYPOCOTYL
LRR-TIR	LEUCINE RICH REPEAT-TOLL INTERLEUKIN 1 RECEPTOR
MADS	MCM1-AGAMOUS-DEFICIENS-SRF
MAPK	MITOGEN-ACTIVATED PROTEIN KINASE
NGS	Next Generation Sequencing
NF-Y	NUCLEAR FACTOR-Y
PTMs	Post-Translational Modifications
SAM	S-ADENOSYL METHIONINE
VPS1	VACUOLAR PROTEIN SORTING 1
WDSAM1	Tryptophan-Aspartic acid-Sterile Alpha Motif 1
