# Supplementary figures and images for: DNA Methylation Analysis of Dormancy Release in Almond (Prunus dulcis) Flower Buds Using Epi-Genotyping by Sequencing
Source: Int J Mol Sci. 2018 Nov 10;19(11):3542. doi: 10.3390/ijms19113542 (PMC6274898; doi:10.3390/ijms19113542)

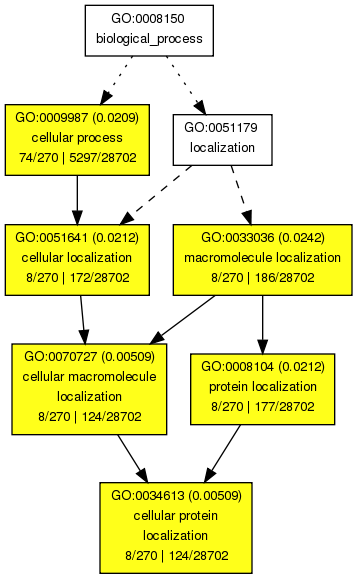

Supplement: Supplementary file 1 [file ijms-19-03542-s001.zip › ijms-369481-R1 Supplementary Material/Prudencio et al. IJMS Fig. S1.tif]

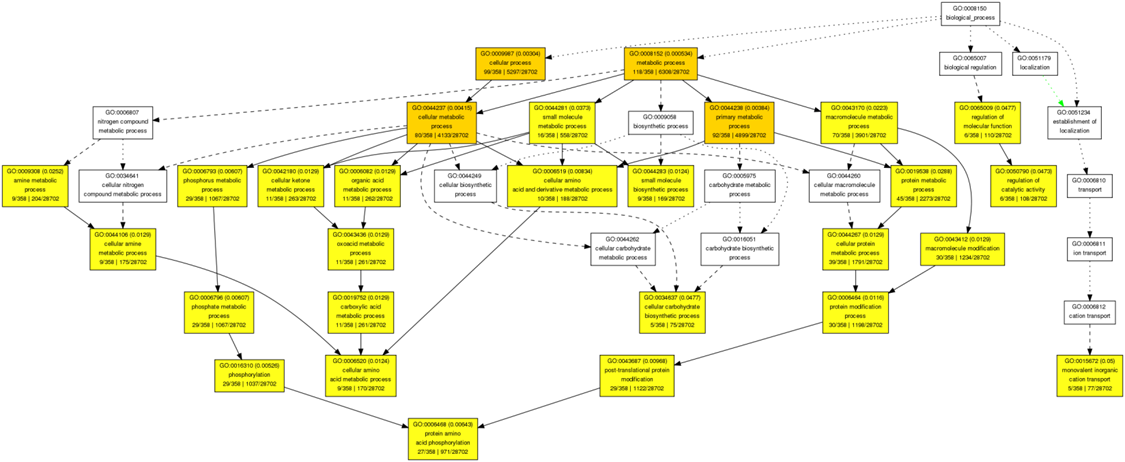

Supplement: Supplementary file 1 [file ijms-19-03542-s001.zip › ijms-369481-R1 Supplementary Material/Prudencio et al. IJMS Fig. S2.tif]
